# Structural Biology Helps Interpret Variants of Uncertain Significance in Genes Causing Endocrine and Metabolic Disorders

**DOI:** 10.1210/js.2018-00077

**Published:** 2018-06-13

**Authors:** Sirawit Ittisoponpisan, Alessia David

**Affiliations:** Structural Bioinformatics Group, Department of Life Sciences, Imperial College London, London, United Kingdom

**Keywords:** disease, genetic variants of uncertain significance, precision medicine, protein structure

## Abstract

**Context:**

Variants of uncertain significance (VUSs) lack sufficient evidence, in terms of statistical power or experimental studies, to allow unequivocal determination of their damaging effect. VUSs are a major burden in performing genetic analysis. Although *in silico* prediction tools are widely used, their specificity is low, thus urgently calling for methods for prioritizing and characterizing variants.

**Objective:**

To assess the frequency of VUSs in genes causing endocrine and metabolic disorders, the concordance rate of predictions from different *in silico* methods, and the added value of three-dimensional protein structure analysis in discerning and prioritizing damaging variants.

**Results:**

A total of 12,266 missense variants reported in 641 genes causing endocrine and metabolic disorders were analyzed. Among these, 4123 (33.7%) were VUSs, of which 2010 (48.8%) were predicted to be damaging and 1452 (35.2%) were predicted to be tolerated according to *in silico* tools. A total of 5383 (87.7%) of 6133 disease-causing variants and 823 (55.8%) of 1474 benign variants were correctly predicted. *In silico* predictions were noninformative in 5.7%, 14.4%, and 16% of damaging, benign, and VUSs, respectively. A damaging effect on 3D protein structure was present in 240 (30.9%) of predicted damaging and 40 (9.7%) of predicted tolerated VUSs (*P* < 0.001). An in-depth analysis of nine VUSs occurring in *TSHR, LDLR, CASR,* and *APOE* showed that they greatly affect protein stability and are therefore strong candidates for disease.

**Conclusions:**

In our dataset, we confirmed the high sensitivity but low specificity of *in silico* predictions tools. 3D protein structural analysis is a compelling tool for characterizing and prioritizing VUSs and should be a part of genetic variant analysis.

## Teaser

3D protein structural analysis helps interpret genetic variants of uncertain clinical significance.

Sequencing projects have yielded thousands of novel genetic variants, and many thousands more are likely to be discovered in the near future as a result of large genetic projects, such as the 100,000 Genomes Project, already under way in the United Kingdom, and a major U.S. initiative aimed at sequencing 1 million individuals. Variants with uncertain significance (VUSs) are a well-known problem among the research community involved in genetic studies. VUSs lack sufficient evidence in terms of statistical power or experimental studies to permit unequivocal determination of their damaging or tolerating effect. VUSs are often identified in patients whose clinical phenotype and family history strongly suggest the presence of a genetic background. However, the lack of clarity on the biological significance of VUSs affects our ability to stratify patients into diagnostic and therapeutic algorithms and hinders screening of their relatives, as well as prenatal screening and counseling.

The magnitude of the problem posed by VUSs is exemplified by the fact that one tenth of variants (24,081 variants of 252,843 unique records with assertion criteria) reported in ClinVar (one the largest international repositories of genetic variants, widely used by the medical and genetics community) [1] are reported as “unclassified” or “conflicting interpretation.”

To understand the functional contribution of genetic variants to the clinical phenotype and response to therapy and, hence, improve diagnostic and therapeutic clinical algorithms, it is crucial to combine recent advances in sequencing technology with *in silico* variant prediction methods, which can help prioritize variants for further *in vitro* testing. Several *in silico* predictive tools have been developed over the years and are widely used by the scientific community for predicting the damaging (or tolerated) effect of genetic variants causing amino acid substitutions. Indeed, genetic variants can affect protein folding and stability, protein function, protein-protein interaction, and protein subcellular localization.

*In silico* prediction methods are typically based on evolutionary conservation alone [*e.g.,* Sorting Tolerant From Intolerant (SIFT)] [2] or in combination with the physico-chemical properties of the residue under investigation (*e.g*., PolyPhen2) [3]. SIFT and PolyPhen2 scores are included in the Ensembl Variant Effect Predictor and are also reported in the Exome Aggregation Consortium (ExAC) database [4]. However, *in silico* predictions suffer from several limitations [5]. In particular, a recent study showed that they tend to overestimate variants with a deleterious effect [6]. The human genome contains an excess of rare variants (those with a frequency <1%) [4,7]. These variants are potential disease candidates when identified in patients. If one considers that there is enrichment in SIFT- and PolyPhen2-predicted damaging variants among the rare compared with common variants [7], it becomes clear that new approaches to prioritize potentially damaging variants are urgently needed.

Endocrine and metabolic disorders have a high incidence and prevalence in the general population [8]. We used the set of genes causing these disorders to assess (1) the frequency of VUSs, (2) the concordance rate of variant predictions from widely used *in silico* methods, and (3) the added value of three-dimensional (3D) protein structure analysis to help discern and prioritize damaging variants that require further *in vitro* testing.

## 1. Materials and Methods

### A. Dataset

We extracted 307,135 human, missense, germline variants from the ClinVar database [1]. ClinVar variant classification was adopted. Variants classified as “variant of unknown significance/suspicious” (those for which there is limited evidence that the variant could be causative of disease) and variants of “unknown significance” (those for which the reported evidence in the literature is incomplete and/or contradictory) where included in the VUS set. An additional 76,520 human missense variants were extracted from UniProt, the databank of proteins (UniProt Release: 2016_05 of May 10, 2016) [9]. In UniProt, variants are classified as “disease_causing” (29,463 variants with evidence of disease association), “polymorphisms” (39,756 variants with no evidence of disease association), and “unclassified” (7301 variants with uncertain implication in disease because evidence against or in favor of a pathogenic role is limited or literature reports are conflicting). The latter were also included in the VUS nonredundant dataset. Endocrine and metabolic disorders were identified by using the International Classification of Diseases, Tenth Revision (ICD-10) and codes obtained from Chapter IV, “Endocrine, Nutritional and Metabolic Diseases (E00-E90),” and from Chapter II, “Malignant Neoplasms of Thyroid and Other Endocrine Glands (C73-C75).” The Online Mendelian Inheritance in Man (OMIM) database [10] was interrogated to identify additional protein-coding genes responsible for endocrine-metabolic disorders. ClinVar, UniProt, OMIM, and ICD-10 entries were cross-referenced to extract all protein-coding genes causing endocrine and metabolic disorders and their unique variants. Only variants that could be mapped onto the canonical protein sequences were studied. When the wild-type residue could not be mapped onto the UniProt protein sequence, cases were excluded from the final dataset. All variants were mapped onto a 3D protein structure (when this was available), as described below.

### B. Variant Predictions

The tolerated or damaging effect of each variant in the dataset was obtained from the following widely used variant predictors: SIFT [2], PolyPhen2 [3], MutationAssessor [11], and Condel [12]. SIFT and MutationAssessor assess the effect of a variant based on its sequence conservation, whereas PolyPhen2 uses sequence conservation and structural features. Condel integrates predictions from two tools, MutationAssessor and FATHMM [13], to generate a new consensus score [12]. The default threshold recommended for each program was used to predict the damaging or tolerated effect of each variant. We applied a binary definition to each variant: damaging vs tolerated. Because PolyPhen2 and MutationAssessor provide multiple effect definitions, we considered the PolyPhen2 predictions “probable deleterious” and “possibly deleterious” and the MutationAssessor predictions “high” and “medium” impact [11] as “damaging,” whereas the PolyPhen2 score “benign” and the MutationAssessor predictions “neutral” and “low” impact as “tolerated.”

### C. Structural Analysis

Experimental structures [x-ray and nuclear magnetic resonance (NMR) coordinates] were extracted from the ProteinDataBank (PDB) [14] and variants were mapped onto structures. In the presence of multiple structures covering the query amino acid position, the best structure (*i.e.,* the one with the highest resolution) was chosen. Structures covering fewer than 100 residues were not used. In the presence of two structures with similar resolution, the one covering the longest amino acid sequence was chosen.

To study the effect of amino acid variation, mutant structures were generated by removing the side chain of the residue under investigation (query residue) and any surrounding residue with at least one atom within a 5Å distance to any atom of the query residue. The mutant side chain of the query residue and the wild-type side chains of the surrounding residues were reintroduced by using the SCWRL4 program [15]. Because this procedure could introduce a bias during the repacking, for each mutant structure we also recreated the wild type-structure. We assessed the validity of this repacking method by comparing the wild-type and corresponding repacked wild-type structures on a set of 606 human, high-quality protein structures (defined as resolution <2.0Å, MolProbity score < 2.0 [16], ≤5% of residues with bond length outliers (>4*σ*), ≤5% of residues with bond angle outliers (>4*σ*), and ≤5% of residues with C*β* deviation outliers (>0.25Å) [17] extracted from the independent dataset of the top 8000 structures (available at http://kinemage.biochem.duke.edu/databases/top8000.php) in the PDB (Supplemental file 606proteins.xls) [18]. Thirty proteins were in common with the set of proteins causing endocrine or metabolic disorders. The average distance between the backbone atoms of the superposed repacked wild-type and mutant structures measured by the root-mean-square deviation (RMSD) was 0.027 ± 0.118 (RMSD mean ± SD). Furthermore, we used this dataset of 606 human protein-coding genes for which high-quality protein structures were available to assess the accuracy of structural analysis in discriminating between deleterious and benign variants.

The structural effect of each VUS was assessed by comparing the properties of the wild-type to the mutant residue by analyzing the structural changes introduced by the mutant residue. In particular, the following structural changes were evaluated: (1) change in solvent accessibility (from buried to exposed or vice-versa), (2) breakage of salt or disulfide bridges, (3) disallowed torsion angles, (4) change in the charge of a buried residue, (5) introduction of a steric clash, (6) replacement of a buried hydrophobic residue with a hydrophilic one, (7) disruption of secondary structure (*i.e.,* introduction of proline in a secondary structure motif, such as an *α* helix), (8) substitution of a buried glycine with any other amino acid, (9) substitution to proline, (10) substitution from glycine to any other residue in the "sharp turn" (as defined by the DSSP program, https://swift.cmbi.umcn.nl/gv/dssp/index.html), and (11) change in cavity volume (cavity filling or cavity expanding).

The following features were considered: (1) salt bridge, defined as at least one pair of atoms on oppositely charge groups within a 5Å distance; (2) disulfide bridge (S-S bridge), defined as the side chains of two cysteines at a 3.3Å distance; (3) disallowed torsion angles, as described in Lovell *et al.* [17]; (4) "hydrophobic" residues (V, I, L, M, F, W, C, A) and hydrophilic residues (R, K, E, D, Q, N) [19]; (5) charged residues (D, E, H, K, R) [20]; (6) secondary structure: helices, strands, and turns were calculated by using DSSP [21; (7) the amino acid percentage relative surface accessibility area calculated by dividing its total surface area with that in the extended conformation (*ϕ* = *Ψ* = 180°) of the Gly–X–Gly tripeptide. Residues were defined as solvent accessible if relative surface accessibility area was ≥9%; otherwise residues were considered buried [22].

A steric clash score was calculated by using the "clashlistcluster" shell script from the MolProbity library. A clash was defined as a van der Waals overlap ≥0.4 Å, and clash score was defined as the average number of clashes per 1000 atoms [18]. We evaluated only local clashes that occurred within a 20Å radius from C*α* of the mutant residue and considered a mutation to be "damaging" when a mutant model had high clash score (≥30) [18] and a ≥70% increase in clash score between mutant and repacked wild-type structures. The cavity volume was calculated by KVFinder [23] by using its default setting. Amino acid substitutions, which affect protein stability, can cause a change in cavity volume that ranges from 5Å^3^ to 150Å^3^ [24–26]. In our independent dataset of 606 human structures, a 70Å^3^ change in cavity volume maximizes the true-positive vs false-positive ratio and significantly discriminates between disease-causing and neutral variants (*χ*^2^ = 23.81; *P* < 0.001).

## 2. Results

We analyzed 641 unique protein-coding genes (Supplemental file 641_genes.txt) causing endocrine and metabolic disorders and harboring 12,266 unique missense variants, of which 4123 (33.7%) were VUSs. These variants occur in genes known to cause endocrine or metabolic disorders and are strong disease-causing candidates when identified in patients with these disorders. Moreover, in our database, 6133 (50%) variants were classified as deleterious (“pathogenic/likely pathogenic” in ClinVar or "disease-causing" in UniProt), 1474 (12%) as benign (“benign or likely benign” in ClinVar and “polymorphisms” in UniProt). The remaining variants were annotated as “risk_factor” (n = 22), “protective” (n = 3), and “involved in drug response” (n = 5). For 495 variants, no clinical significance was reported in ClinVar.

### A. *In silico* Prediction Tools Overestimate the Damaging Effect of “Neutral” Variants

*In silico* predictions for variants classified as benign and deleterious were analyzed: 5383 of 6133 (87.7%) deleterious variants were correctly identified as damaging by all or most *in silico* predictors, and 823 of 1474 (55.8%) benign variants were correctly predicted to be tolerated (Table 1). We thereafter analyzed *in silico* predictions for 4123 VUSs: 2010 (48.8%) were predicted to be damaging and 1452 (35.2%) to be tolerated. For the remaining VUSs, *in silico* results were noninformative because there were equal numbers of “damaging” and “tolerated” predictions for each variant (Table 1).

**Table 1.** *In silico* Predictions for Genetic Variants Identified in Genes Causing Endocrine and Metabolic Disorders

Clinical Significance	*In silico* Predictions, n (%)			Total, n
	Damaging	Tolerated	Noninformative	
Disease-causing	5383 (87.8)	403 (6.6)	347 (5.7)	6133
Benign	439 (29.8)	823 (55.8)	212 (14.4)	1474
VUS	2011 (48.9)	1452 (35.1)	661 (16)	4124

Predictions were classified as “noninformative” in the presence of an equal number of “damaging” and “tolerated” responses from different software for the same variant (*e.g.*, variant X predicted to be “damaging” by SIFT and Condel and “tolerated” by PolyPhen2 and MutationAssessor).

*In silico* predictions tools thus appear to have a high sensitivity and a low specificity, at least for variants occurring in genes known to cause endocrine and metabolic disorders. Although our results cannot at present be generalized to the whole proteome, our findings confirm what was previously reported on a smaller dataset of variants in genes causing epilepsy syndromes [27].

### B. Structural Predictions for VUSs

We assessed the added value of 3D structural analysis in prioritizing VUSs, based on their damaging effect on protein structure (Supplemental file VUS_predictions.xls). First, we tested whether structural analysis was able to differentiate between disease-causing and benign variants on an independent dataset of 606 human proteins, for which a high-quality 3D structure (see Materials and Methods) was available. One or more possibly damaging structural changes were present in 851 of 1990 deleterious variants, compared with 361 of 2280 benign variants (*χ*^2^ test, *P* < 0.001), thus suggesting that 3D structural analysis is informative in discriminating between damaging and neutral variants.

We then performed structural analysis on the impact on protein structure of the 4123 VUSs from our dataset of proteins involved in endocrine and metabolic disorders and compared the results with predictions from widely used *in silico* tools, namely SIFT, PolyPhen2, MutationAssessor, and Condel (Table 2). The 4123 VUSs were distributed among 247 proteins. We examined the 365,464 structural files (x-ray and NMR coordinates) deposited in the PDB. At least one structural file was available for 118 (47.8%) proteins, and we were able to map and perform structural analysis for 1389 VUSs (number of PDB files analyzed: 162). 3D structural analysis could be performed in 776 (38.6%) of 2010 variants that were predicted to be damaging by all or most *in silico* predictors and in 410 (28.2%) of 1452 variants that were predicted to be tolerated according to all or most *in silico* predictors. A damaging effect on protein structure stability was more likely to be observed in VUSs predicted to be damaging rather than tolerated (240 vs 40; *P* < 0.001) (Table 2). Interestingly, variants with a full, rather than partial, concordance on their damaging effect were also more likely to cause structural damage (182 vs 58 variants; *P* < 0.01). However, this was not observed for variants predicted to be tolerated (*P* = 0.43).

**Table 2.** Results for VUSs From *In Silico* Prediction Tools and From 3D Structural Analysis

Prediction	Total VUSs Analyzed, n	VUSs Mapped Onto 3D Structure, n (%)	Effect on 3D Structure, n (%)	
			Damaging VUSs	Tolerated VUSs
Damaging				
By all predictors	1343	536 (39.9)	182 (34)	354 (66)
By most predictors	667	240 (36.0)	58 (24.1)	182 (75.8)
Total	2010	776 (38.6)	240 (30.9)	536 (69.1)
Noninformative				
Equal number of “damaging” and “tolerated” predictions	661	203 (30.7)	27 (13.3)	176 (85.7)
Tolerated				
By all predictors	819	227 (27.7)	24 (10.6)	203 (89.4)
By most predictors	633	183 (28.9)	16 (8.7)	167 (91.3)
Total	1452	410 (28.2)	40 (9.7)	370 (90.3)

In 661 VUSs (16%), there were equal numbers of damaging and tolerated *in silico* predictions for each variant (noninformative predictions). 3D structural analysis could be performed on 203 variants (30.7%), with a damaging effect on protein structure stability observed in 27 (13.3%) VUSs.

Overall, there was agreement between 3D structural analysis and *in silico* predictions, and our results demonstrated the added value of 3D structure analysis in identifying the VUSs that are likely to be nontolerated and that may require validation by *in vitro* experiments or by genetic confirmation on other affected family members.

## 3. Description of Cases

Manual in-depth analysis of VUSs that had several damaging structural features suggesting a deleterious effect was performed (Supplemental Table S1).

### A. Thyroid-Stimulating Hormone Receptor

Thyroid-stimulating hormone receptor (TSHR) is a G protein–coupled receptor that plays a crucial role in thyroid hormone metabolism. Mutations in this gene have been identified in several widely spread thyroid conditions, such as autonomous toxic thyroid adenomas, Graves disease, and autoimmune hypothyroidism [28].

*p.Gly132Arg* (rs760874290) is reported in ClinVar under “conflicting interpretations of pathogenicity” because it has been deposited by three different submitters as “benign,” “likely pathogenic,” and of “uncertain significance.” A literature search showed that p.Gly132Arg has been identified in compound heterozygosity with p.Arg450His in two patients with congenital hypothyroidism: a 14-year-old Japanese patient [pretreatment thyroid-stimulating hormone (TSH) level, 18 mU/L (normal range, 0.5 to 5.0 mU/L); free T4, 1.6 ng/dL (normal range, not available); mild thyroid hypoplasia] and a 16-year-old Korean girl (pretreatment TSH levels, 16.0 mU/L; normal thyroid on ultrasonography; 99mTc uptake, 0%) [29]. p.Gly132Arg was also identified in single heterozygosity in an 11-year-old Korean girl [pretreatment TSH level, 41.5 mU/liter; normal thyroid on ultrasonography; reduced 99mTc uptake at 1.7% (normal uptake, 2.5% to 7%)] [30]. *In silico* predictions for p.Gly132Arg were as follows: SIFT score, tolerated; MutationAssessor score, neutral; Condel score, neutral; PolyPhen2 score, probably damaging.

Structural analysis was performed by using the crystal structure of the extracellular domain of the human TSHR (PDB ID: 2XWT, 1.9 Å). Gly132 is located in the extracellular leucine-rich repeat (LRR) domain of the TSHR. The large concave surface of the TSHR LRR represents the site of interaction between TSHR and TSH, as well as TSHR and its autoantibodies [31]. Glycine 132 is located on the *β* strand of the fifth LRR repeat, between residues Phe130 and Phe134, which interact with Lys91 and Tyr88, respectively, in the TSH–TSHR model complex (and with Pro97 in the thyroid-stimulating autoantibody M22–TSHR complex) [32]. Our structural analysis shows that replacement of the small and neutral Gly132 with the large and charged side chain of arginine is likely to induce an important conformational change, which could displace Phe130, thus altering the ability of TSHR to bind TSH, as well as TSHR autoantibodies. An extensive literature search revealed that the TSHR-Gly132Arg mutant generated *in vitro* has normal expression levels. Competitive TSH-binding studies showed absent binding activity and a cAMP response of 26% compared with wild-type; these findings support the results of our analysis, which suggest that the Gly-to-Arg substitution results in functional TSHR impairment rather protein misfolding [29].

### B. Apolipoprotein E

Liver-derived apolipoprotein E (APOE) is a main ligand for low-density lipoprotein (LDL) receptor (LDLR) and has a crucial role in removing from circulation triglyceride-rich lipoproteins, which are a major risk factor for ischemic heart disease. APOE has also a major role in brain lipid metabolism, and the APOE *ε*4 isoform is a well-established risk factor for Alzheimer’s disease [33].

*p.Leu46Pro* (rs769452) is reported in ClinVar under “uncertain significance.” This variant was first reported in a 6-year-old girl with an LDL cholesterol level of 1.97 g/L, a high-density lipoprotein cholesterol level of 0.70 g/L, a triglyceride level of 0.85 g/L, and a strong history of familial hypercholesterolemia (the lipid profile of the patient's affected mother, who was a carrier of this mutation, is not available) [34]. Of note, this variant has also been associated with an increased risk for late-onset Alzheimer disease [35]. *In silico* predictions are as follows: SIFT, tolerated; MutationAssessor, medium effect; PolyPhen2, possibly damaging; Condel, damaging. Structural analysis was performed using the NMR solution structure of the human APOE receptor–binding domain (PDB ID: 2KC3, 1.9 Å). Leu46 is located in an *α* helix, and substitution to proline is likely to induce a bend in the helix, which can greatly destabilize the APOE structure and lead to protein misfolding. A literature search revealed that the effect of this variant on the structure of APOE isoform 4 (APOE *ε*4, Leu28Pro) was studied *in vitro.* The mutant protein was thermodynamically unstable and resulted in early degradation [36].

### C. Calcium-Sensing Receptor

Calcium-sensing receptor (CASR) is a family C G protein–coupled receptor expressed on the cell surface of tissues involved in calcium homeostasis. It senses perturbations in extracellular calcium levels and modulates parathyroid hormone secretion and renal Ca^2+^ excretion accordingly [37]. *CASR*-inactivating mutations result in familial hypocalciuric hypercalcemia when in heterozygosity, and severe neonatal hyperparathyroidism when in homozygosity [38, 39].

*p.Gly36Arg* (rs193922420) is reported in ClinVar under “conflicting interpretation of pathogenicity.” The condition associated with this variant was familial hypocalciuric hypercalcemia, although no clinical data were available. This variant is predicted to be damaging by all *in silico* predictors. Our structural analysis (PDB ID: 5FBK, 2.1 Å) showed that Gly36 is part of a *β* strand in the extracellular domain (ECD) domain, close to a CA^2+^ binding site. Substitution of glycine to the large, charged arginine is predicted to cause a steric clash and disrupt CASR’s protein structure.

**Figure 1.**
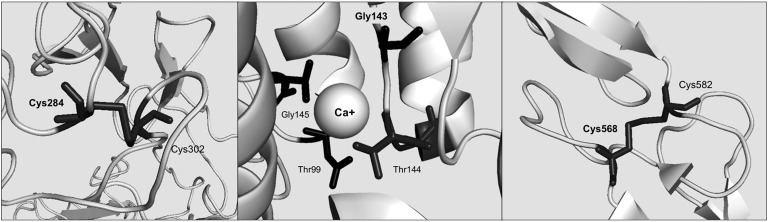
Structural analysis of VUSs occurring in *LDLR* and *CASR*. (Left) p.Cys284Ser abolishes a cysteine bond in the LDLR protein. (Middle) p.Gly143Arg may disrupt Ca^+^ (presented as a sphere) binding site in CASR protein. (Right) p.Cys568Gly is likely to disrupt a disulfide bridge in the CASR protein.

*p.Gly143Arg* (rs769256610) was identified in heterozygosity in a 65-year-old woman with familial hypocalciuric hypercalcemia. Her laboratory data were as follows: total calcium, 10.7 mg/dL (normal range, 8.5 to 10.5 mg/dL); ionized calcium, 5.68 mg/dL (normal range, 4.48 to 5.28 mg/dL); phosphate, 3.0 mg/dL (normal range, 2.2 to 4.1 mg/dL); parathyroid hormone, 40 ng/L (normal range, 10 to 65 ng/L); 25-hydroxyvitamin D, 38 ng/mL (normal range, 9.0 to 52.0 ng/mL); 24-hour urine calcium, 40 mg (normal range, 50 to 250 mg). Spine T score was −2.3 SD, indicating low bone mineral density. Her brother also had mild hypercalcemia (values not reported). His *CASR* genotype was not reported. This variant is reported in ClinVar under “uncertain significance” and is predicted to be damaging by all *in silico* predictors. Structural analysis was performed by using the experimental crystal structure of the extracellular homodimer domain of CASR (PDB ID: 5K5T, 3.1 Å). Gly143 is in the short loop connecting a *β* sheet with an *α* helix of the LB1 domain of CASR. Moreover, Gly143 is close to one of the four Ca^2+^ binding sites. Substitution of the glycine with arginine is likely to cause a steric clash, thus destabilizing the CASR structure (Fig. 1). Interestingly, substitution of Gly143 with glutamic acid has been demonstrated to lead to a reduction in the amount of mature glycosylated CASR, with little or no response to Ca^2+^ [40]. We postulate that substitution of Gly143 with arginine will have a similar effect on CASR structure and function.

*p.Cys568Gly* (rs1060502851) is reported in ClinVar under “uncertain significance.” The conditions associated with this variant are familial hypocalciuric hypercalcemia and autosomal-dominant hypocalcemia. No clinical data on the clinical phenotype were available. *In silico* predictions for this variant are as follows: PolyPhen2, benign; SIFT, damaging; Condel, damaging; MutationAssessor, not available. Structural analysis was performed by using the crystal structure of the human CASR extracellular domain (PDB ID: 5K5T, 3.1 Å) and showed that Cys568 forms a disulfide bridge with Cys582. Substitution of Cys568 with any other amino acid disrupts this crucial structural element, thus causing structural damage to CASR (Fig. 1). No *in vitro* functional studies are available on the effect of this variant. However, other amino acid substitutions of Cys582 (p.Cys582Tyr and p.Cys582Phe), which are likely to disrupt the same disulfide bridge, have been shown to cause familial hypocalciuric hypercalcemia, neonatal severe hyperparathyroidism, and autosomal-dominant hypocalcemia.

### D. Low-Density Lipoprotein Receptor

The LDLR is an important contributor to the removal of LDL cholesterol from the circulation. Deleterious genetic variants in this gene are responsible for familial hypercholesterolemia (FH). Heterozygous FH, which has a prevalence of approximately 1:200 in some European populations, is an important risk factor for premature cardiovascular disease [41]. More than 1000 genetic variations have been detected in this gene so far. There is great interest in understanding which *LDLR* variants affect LDLR because this can aid in the identification of individuals at increased risk for premature cardiovascular disease.

*p.Cys284Ser* (rs879254693) is reported under “uncertain significance” in ClinVar. It was described in patients from the Norway FH cohort [42] and is predicted to be damaging by SIFT and Condel and tolerated by PolyPhen2. Structural analysis was performed by using the crystal structure of the human LDLR/proprotein convertase subtilisin/kexin type 9 complex (PDB ID: 3P5C, 4.2 Å). Cys284 forms a cysteine bond with Cys302. Substitution with serine disrupts this bond because it is likely to cause structural damage to the LDLR protein structure (Fig. 1).

*p.Cys329Tyr* (rs761954844) is located in the epidermal growth factor–like 1 domain of LDLR. It is classified as of “conflicting interpretation of pathogenicity” in ClinVar because it has been annotated as “likely benign” as well as “likely pathogenic” and “pathogenic.” This variant was first reported in two unrelated Chinese patients with a lipid profile and family history suggesting FH. Moreover, it was shown to cosegregate with the FH phenotype in affected relatives of both patients [43]. This variant has since been reported in several patients with possible heterozygous FH from different ethnic background [44–46]. *In silico* predictions suggest a deleterious effect of this variant (PolyPhen2, Condel, and SIFT; no prediction available from MutationAssessor). Structural analysis was performed by using the crystal structure of the extracellular domain of the LDLR (PDB ID: 1N7D, 3.7 Å). Cys329 forms a cysteine bond with Cys318, thus helping to stabilize the LDLR structure. Substitution with tyrosine disrupts such a bridge, thus causing structural instability. Indeed, previous *in vitro* studies have shown a marked reduction in the mutant LDLR expression on the cell surface and its ability to bind and internalize LDL [47].

*p.Ser499Pro* (rs879254921) is reported under “conflicting interpretation of pathogenicity” in ClinVar because it was reported as “likely benign,” as well as “likely pathogenic.” Ser499 occurs in the ligand-binding region (epidermal growth factor precursor homology domains) of LDLR. *In silico* predictions are as follows: PolyPhen2, benign; MutationAssessor, medium effect; SIFT, damaging; Condel, damaging. Structural analysis was performed by using the crystal structure of the human LDLR (PDB ID: 1IJQ, 1.5 Å) and revealed that Ser499 is located within an *α* helix. Its substitution with proline is likely to cause a bend in the *α* helix, thus causing major disruption to the LDLR structure.

*p.Cys698Trp* (rs879255137) is reported in ClinVar under “conflicting interpretation of pathogenicity” because it has been reported as both “likely pathogenic“ and of “uncertain significance” in patients with FH (no clinical data available). *In silico* predictors suggest a damaging effect. Structural analysis was performed by using the crystal structure of the human LDLR (PDB ID: 1IJQ, 1.5 Å) and revealed that Cys698 forms a disulfide bond with Cys711. Substitution of Cys698 with any other residue disrupts this structurally important bond.

## 4. Discussion

In this study, we quantified the burden of VUSs in genes causing endocrine and metabolic disorders and showed that they represent a third of known variants in these genes. Personalized medicine relies on the ability to identify actionable genetic variants (*i.e.,* variants that influence a patient’s response to treatment or prognosis) [48]. For example, patients with heterozygous FH have a higher cardiovascular risk than the general population, and such a genetic diagnosis dictates a more aggressive treatment of hypercholesterolemia compared with patients with simple dyslipidemia [49].

The first step toward the goal of implementing actionable genetic variations into clinical practice is the identification of variations, which affect phenotype. Missense variants (*i.e.,* those leading to an amino acid change) are compelling candidates because they can directly modify protein structure and/or function. A large proportion of human genetic variations are missense variants, and identification of those of clinical significance remains a major challenge in modern genetics. Several variant effect predictors trained on large datasets of neutral and damaging variations occurring across the whole proteome have been developed over the years. However, their specificity remains low [50,51]. In our dataset, 88% of known damaging variants and almost half of known benign variants were predicted to be damaging by some of the most widely used *in silico* predictions tools.

Other authors have reported a high sensitivity but low specificity for *in silico* tools used to assess the effect of variants in disease-causing genes involved in the pathogenesis of epileptic encephalopathies [27]. Recently, *in vitro* testing of variants identified in the cancer gene *TP53* and predicted to be damaging by *in silico* tools revealed that only half of the variants affected TP53 activity [6]. Overprediction of potentially damaging variants generates a high number of genetic variations that require further *in vitro* testing, an expensive and time-consuming process. Therefore, approaches to filtering genetic variants are urgently needed.

In this study, we used 3D structural analysis to assess the impact of VUSs on protein function and structure stability. We first assessed the utility of this method on an independent dataset of proteins and demonstrated that it accurately identifies enrichment of damaging structural effects among known deleterious variants, compared with benign variants. We then applied this approach to filter VUSs. Although structural analysis was possible for only one third of VUSs in our dataset, there was concordance between *in silico* predictions and structural analysis. Furthermore, we were able to identify a subset of predicted damaging VUSs, which are potentially disease-causing. The relatively low number of VUSs that could be structurally analyzed reflects the still limited number of proteins for which a good-quality experimental 3D structure is available.

A major international effort is ongoing to solve the experimental structure of clinically important proteins, as demonstrated by the steadily growing availability of structures in PDB, from 23,000 in 2003 to the current >133,000. Although an experimental 3D structure is available only for less than a third (6064) of human proteins (and in most cases, it covers only part of the protein), the structural coverage of the human proteome can be greatly enhanced by using homology modeling. Although the use of homology modeling to understand the impact of variants on protein structure is successfully used [52,53], it requires in-depth manual curation and cannot be performed by an automated process. For this reason, in this study, we chose to perform a structural analysis only in cases for which a 3D structure was available, thus limiting the number of VUSs that could be analyzed.

Our case studies demonstrate the value of structural analysis in helping us understand the molecular mechanisms by which a variant can affect phenotype and cause disease, for example, by disrupting structurally important bonds and destabilizing protein structure. Such information cannot be obtained by prediction tools, such as PolyPhen2, SIFT, or CADD, which return only a binary response: damaging or tolerated. Understanding how a genetic variant affects phenotype is essential in guiding *in vitro* experiments [54], especially in the presence of VUSs.

Our case studies also highlight the importance of building gene and disease-specific databases, with curated detailed information on genetic variants. Many of our case studies were variants, which have been deposited in ClinVar or other publicly repositories as both benign and pathogenic. Through extensive literature search, we were able to obtain additional information on these variants and, in some cases, retrieved experimental data that validated our structural predictions. This is an enormously time-consuming process that requires manual curation. Curated databases that allow automatic retrieval of variant and patient information will become invaluable when initiatives, such as the 100,000 Genome Project, release a vast amount of novel genetic data that require interpretation.

A limitation of this study is that we chose to perform structural analysis only on VUSs and not on the entire set of benign and damaging variants in genes causing endocrine and metabolic disorders. Instead, we chose to test our structural approach on a dataset of benign and damaging variants in a different dataset of proteins (not linked to endocrine or metabolic disorders), to avoid potential bias. Another limitation is that we performed structural analysis on monomeric proteins and did not assess the impact of amino acid substitutions on protein-protein interactions. Protein interaction sites are a hot spot for damaging variants [55]. Identification of VUSs that affect protein interaction and potentially disrupt biological pathways is part of future work, as it can also assist in guiding *in vitro* studies [54]. Our ability to understand which biological pathway is affected by a variant is important, especially because several proteins causing endocrine and metabolic disorders are pleiotropic and can thus cause different disorders according to where in the protein they occur [56].

In conclusion, VUSs are an important burden in our understanding of the genetic basis of endocrine and metabolic disorders and in the identification of actionable variants. Although the use of multiple *in silico* variant predictors may be helpful in prioritizing potentially damaging variants for further *in vitro* testing, in-depth understanding of the molecular mechanisms of the disease under investigation and the structure, function, and biological network of the proteins involved in its pathogenesis remains crucial. Protein structural analysis is a compelling tool for prioritizing genetic variants and should be used more extensively, especially for assessing VUSs.

## Supplementary Material

Supplemental Table S1

Supplemental Data 1

Supplemental Data 2

Supplemental Data 3

Supplemental Data 4

## Abbreviations:

3D: three-dimensional
APOE: apolipoprotein E
CASR: calcium-sensing receptor
FH: familial hypercholesterolemia
ICD-10: International Classification of Diseases, Tenth Revision
LDL: low-density lipoprotein
LDLR: low-density lipoprotein receptor
LRR: leucine-rich repeat
NMR: nuclear magnetic resonance
OMIM: Online Mendelian Inheritance in Man
PDB: ProteinDataBank
SIFT: Sorting Tolerant From Intolerant
TSH: thyroid-stimulating hormone
TSHR: thyroid-stimulating hormone receptor
VUS: variant of uncertain significance

## References

[1] Landrum MJ, Lee JM, Benson M, Brown G, Chao C, Chitipiralla S, Gu B, Hart J, Hoffman D, Hoover J, Jang W, Katz K, Ovetsky M, Riley G, Sethi A, Tully R, Villamarin-Salomon R, Rubinstein W, Maglott DR ClinVar: public archive of interpretations of clinically relevant variants. Nucleic Acids Res. 2016;44(D1):D862–D868. 26582918 10.1093/nar/gkv1222 PMC4702865

[2] Kumar P, Henikoff S, Ng PC Predicting the effects of coding non-synonymous variants on protein function using the SIFT algorithm. Nat Protoc. 2009;4(7):1073–1081. 19561590 10.1038/nprot.2009.86

[3] Adzhubei I, Jordan DM, Sunyaev SR Predicting functional effect of human missense mutations using PolyPhen-2. Curr. Protoc. Hum. Genet. 2013;(SUPPL.76). 10.1002/0471142905.hg0720s76 PMC4480630 23315928

[4] Lek M, Karczewski KJ, Minikel EV, Samocha KE, Banks E, Fennell T, O’Donnell-Luria AH, Ware JS, Hill AJ, Cummings BB, Tukiainen T, Birnbaum DP, Kosmicki JA, Duncan LE, Estrada K, Zhao F, Zou J, Pierce-Hoffman E, Berghout J, Cooper DN, Deflaux N, DePristo M, Do R, Flannick J, Fromer M, Gauthier L, Goldstein J, Gupta N, Howrigan D, Kiezun A, Kurki MI, Moonshine AL, Natarajan P, Orozco L, Peloso GM, Poplin R, Rivas MA, Ruano-Rubio V, Rose SA, Ruderfer DM, Shakir K, Stenson PD, Stevens C, Thomas BP, Tiao G, Tusie-Luna MT, Weisburd B, Won H-H, Yu D, Altshuler DM, Ardissino D, Boehnke M, Danesh J, Donnelly S, Elosua R, Florez JC, Gabriel SB, Getz G, Glatt SJ, Hultman CM, Kathiresan S, Laakso M, McCarroll S, McCarthy MI, McGovern D, McPherson R, Neale BM, Palotie A, Purcell SM, Saleheen D, Scharf JM, Sklar P, Sullivan PF, Tuomilehto J, Tsuang MT, Watkins HC, Wilson JG, Daly MJ, MacArthur DG; Exome Aggregation Consortium Analysis of protein-coding genetic variation in 60,706 humans. Nature. 2016;536(7616):285–291. 27535533 10.1038/nature19057 PMC5018207

[5] Kassahn KS, Scott HS, Caramins MC Integrating massively parallel sequencing into diagnostic workflows and managing the annotation and clinical interpretation challenge. Hum Mutat. 2014;35(4):413–423. 24510514 10.1002/humu.22525

[6] Miosge LA, Field MA, Sontani Y, Cho V, Johnson S, Palkova A, Balakishnan B, Liang R, Zhang Y, Lyon S, Beutler B, Whittle B, Bertram EM, Enders A, Goodnow CC, Andrews TD Comparison of predicted and actual consequences of missense mutations. Proc Natl Acad Sci USA. 2015;112(37):E5189–E5198. 26269570 10.1073/pnas.1511585112 PMC4577149

[7] Marth GT, Yu F, Indap AR, Garimella K, Gravel S, Leong WF, Tyler-Smith C, Bainbridge M, Blackwell T, Zheng-Bradley X, Chen Y, Challis D, Clarke L, Ball EV, Cibulskis K, Cooper DN, Fulton B, Hartl C, Koboldt D, Muzny D, Smith R, Sougnez C, Stewart C, Ward A, Yu J, Xue Y, Altshuler D, Bustamante CD, Clark AG, Daly M, DePristo M, Flicek P, Gabriel S, Mardis E, Palotie A, Gibbs R; 1000 Genomes Project The functional spectrum of low-frequency coding variation. Genome Biol. 2011;12(9):R84. 21917140 10.1186/gb-2011-12-9-r84 PMC3308047

[8] Golden SH, Robinson KA, Saldanha I, Anton B, Ladenson PW Clinical review: Prevalence and incidence of endocrine and metabolic disorders in the United States: a comprehensive review. J Clin Endocrinol Metab. 2009;94(6):1853–1878. 19494161 10.1210/jc.2008-2291 PMC5393375

[9] The UniProt Consortium UniProt: the universal protein knowledgebase. Nucleic Acids Res. 2017;45(D1):D158–D169. 27899622 10.1093/nar/gkw1099 PMC5210571

[10] Amberger JS, Hamosh A Searching Online Mendelian Inheritance in Man (OMIM): a knowledgebase of human genes and genetic phenotypes. Curr Protoc Bioinforma. 2017;58:1.2.1–1.2.12. 10.1002/cpbi.27 PMC5662200 28654725

[11] Reva B, Antipin Y, Sander C Predicting the functional impact of protein mutations: application to cancer genomics. Nucleic Acids Res. 2011;39(17):e118. 21727090 10.1093/nar/gkr407 PMC3177186

[12] González-Pérez A, López-Bigas N Improving the assessment of the outcome of nonsynonymous SNVs with a consensus deleteriousness score, Condel. Am J Hum Genet. 2011;88(4):440–449. 21457909 10.1016/j.ajhg.2011.03.004 PMC3071923

[13] Shihab HA, Gough J, Cooper DN, Stenson PD, Barker GLA, Edwards KJ, Day INM, Gaunt TR Predicting the functional, molecular, and phenotypic consequences of amino acid substitutions using hidden Markov models. Hum Mutat. 2013;34(1):57–65. 23033316 10.1002/humu.22225 PMC3558800

[14] Berman H, Henrick K, Nakamura H Announcing the worldwide Protein Data Bank. Nat Struct Biol. 2003;10(12):980. 14634627 10.1038/nsb1203-980

[15] Krivov GG, Shapovalov MV, Dunbrack RL Jr Improved prediction of protein side-chain conformations with SCWRL4. Proteins. 2009;77(4):778–795. 19603484 10.1002/prot.22488 PMC2885146

[16] Williams CJ, Headd JJ, Moriarty NW, Prisant MG, Videau LL, Deis LN, Verma V, Keedy DA, Hintze BJ, Chen VB, Jain S, Lewis SM, Arendall WB, Snoeyink J, Adams PD, Lovell SC, Richardson JS, Richardson DC MolProbity: More and better reference data for improved all-atom structure validation. Protein Sci. 2017;27:293–315. 10.1002/pro.3330 PMC5734394 29067766

[17] Lovell SC, Davis IW, Adrendall WB, de Bakker PIW, Word JM, Prisant MG, Richardson JS, Richardson DC Structure validation by C alpha geometry: phi, psi and C beta deviation. Proteins. 2002;2003(50):437–450. 10.1002/prot.10286 12557186

[18] Hintze BJ, Lewis SM, Richardson JS, Richardson DC Molprobity’s ultimate rotamer-library distributions for model validation. Proteins. 2016;84(9):1177–1189. 27018641 10.1002/prot.25039 PMC4983197

[19] Pommi ÈC, Levadoux Sè, Sabatier R, Lefranc G, Lefranc MP. IMGT standardized criteria for statistical analysis of immunoglobulin V-Region amino acid properties. J Mol Recognit. 2004;17:17–32. 14872534 10.1002/jmr.647

[20] Betts MJ, Russell RB. Amino acid properties and consequences of subsitutions. In: Barnes MR, Gray IC, eds. Bioinformatics for Geneticists. West Sussex: John Wiley; 2003:289–316.

[21] Kabsch W, Sander C Dictionary of protein secondary structure: pattern recognition of hydrogen-bonded and geometrical features. Biopolymers. 1983;22(12):2577–2637. 6667333 10.1002/bip.360221211

[22] Rost B, Sander C Conservation and prediction of solvent accessibility in protein families. Proteins. 1994;20(3):216–226. 10.1002/prot.340200303 7892171

[23] Oliveira SHP, Ferraz FAN, Honorato RV, Xavier-Neto J, Sobreira TJP, de Oliveira PSL KVFinder: steered identification of protein cavities as a PyMOL plugin. BMC Bioinformatics. 2014;15(1):197. 24938294 10.1186/1471-2105-15-197 PMC4071799

[24] Eriksson AE, Baase WA, Zhang XJ, Heinz DW, Blaber M, Baldwin EP, Matthews BW Response of a protein structure to cavity-creating mutations and its relation to the hydrophobic effect. Science. 1992;255(5041):178–183. 1553543 10.1126/science.1553543

[25] Xu J, Baase WA, Baldwin E, Matthews BW The response of T4 lysozyme to large-to-small substitutions within the core and its relation to the hydrophobic effect. Protein Sci. 1998;7(1):158–177. 9514271 10.1002/pro.5560070117 PMC2143816

[26] Buckle AM, Cramer P, Fersht AR Structural and energetic responses to cavity-creating mutations in hydrophobic cores: observation of a buried water molecule and the hydrophilic nature of such hydrophobic cavities. Biochemistry. 1996;35(14):4298–4305. 8605178 10.1021/bi9524676

[27] Holland KD, Bouley TM, Horn PS Comparison and optimization of in silico algorithms for predicting the pathogenicity of sodium channel variants in epilepsy. Epilepsia. 2017;58(7):1190–1198. 28518218 10.1111/epi.13798 PMC5505324

[28] Tuncel M Thyroid stimulating hormone receptor. Mol Imaging Radionucl Ther. 2017;26(Suppl 1):87–91. 28117293 10.4274/2017.26.suppl.10 PMC5283706

[29] Narumi S, Muroya K, Abe Y, Yasui M, Asakura Y, Adachi M, Hasegawa T TSHR mutations as a cause of congenital hypothyroidism in Japan: a population-based genetic epidemiology study. J Clin Endocrinol Metab. 2009;94(4):1317–1323. 19158199 10.1210/jc.2008-1767

[30] Lee S-T, Lee DH, Kim J-Y, Kwon M-J, Kim J-W, Hong Y-H, Lee Y-W, Ki C-S Molecular screening of the TSH receptor (TSHR) and thyroid peroxidase (TPO) genes in Korean patients with nonsyndromic congenital hypothyroidism. Clin Endocrinol (Oxf). 2011;75(5):715–721. 21707688 10.1111/j.1365-2265.2011.04156.x

[31] Núñez Miguel R, Sanders J, Furmaniak J, Smith BR Structure and activation of the TSH receptor transmembrane domain. Auto Immun Highlights. 2017;8(1):2. 27921237 10.1007/s13317-016-0090-1 PMC5136658

[32] Núñez Miguel R, Sanders J, Chirgadze DY, Furmaniak J, Rees Smith B Thyroid stimulating autoantibody M22 mimics TSH binding to the TSH receptor leucine rich domain: a comparative structural study of protein-protein interactions. J Mol Endocrinol. 2009;42(5):381–395. 19221175 10.1677/JME-08-0152

[33] Rasmussen KL Plasma levels of apolipoprotein E, APOE genotype and risk of dementia and ischemic heart disease: a review. Atherosclerosis. 2016;255:145–155. 28340945 10.1016/j.atherosclerosis.2016.10.037

[34] Wintjens R, Bozon D, Belabbas K, MBou F, Girardet JP, Tounian P, Jolly M, Boccara F, Cohen A, Karsenty A, Dubern B, Carel JC, Azar-Kolakez A, Feillet F, Labarthe F, Gorsky AM, Horovitz A, Tamarindi C, Kieffer P, Lienhardt A, Lascols O, Di Filippo M, Dufernez F Global molecular analysis and APOE mutations in a cohort of autosomal dominant hypercholesterolemia patients in France. J Lipid Res. 2016;57(3):482–491. 26802169 10.1194/jlr.P055699 PMC4766997

[35] Corder EH, Saunders AM, Strittmatter WJ, Schmechel DE, Gaskell PC, Small GW, Roses AD, Haines JL, Pericak-Vance MA Gene dose of apolipoprotein E type 4 allele and the risk of Alzheimer’s disease in late onset families. Science. 1993;261(5123):921–923. 8346443 10.1126/science.8346443

[36] Argyri L, Dafnis I, Theodossiou TA, Gantz D, Stratikos E, Chroni A Molecular basis for increased risk for late-onset Alzheimer disease due to the naturally occurring L28P mutation in apolipoprotein E4. J Biol Chem. 2014;289(18):12931–12945. 24644280 10.1074/jbc.M113.538124 PMC4007480

[37] Riccardi D, Kemp PJ The calcium-sensing receptor beyond extracellular calcium homeostasis: conception, development, adult physiology, and disease. Annu Rev Physiol. 2012;74(1):271–297. 22017175 10.1146/annurev-physiol-020911-153318

[38] Chakravarti B, Chattopadhyay N, Brown EM Signaling through the extracellular calcium-sensing receptor (CaSR). Adv Exp Med Biol. 2012;740:103–142. 22453940 10.1007/978-94-007-2888-2_5

[39] Brown EM Role of the calcium-sensing receptor in extracellular calcium homeostasis. Best Pract Res Clin Endocrinol Metab. 2013;27(3):333–343. 23856263 10.1016/j.beem.2013.02.006

[40] Bai M, Quinn S, Trivedi S, Kifor O, Pearce SH, Pollak MR, Krapcho K, Hebert SC, Brown EM Expression and characterization of inactivating and activating mutations in the human Ca2+o-sensing receptor. J Biol Chem. 1996;271(32):19537–19545. 8702647 10.1074/jbc.271.32.19537

[41] Sharifi M, Futema M, Nair D, Humphries SE Genetic architecture of familial hypercholesterolaemia. Curr Cardiol Rep. 2017;19(5):44. 28405938 10.1007/s11886-017-0848-8 PMC5389990

[42] Leren TP, Manshaus T, Skovholt U, Skodje T, Nossen IE, Teie C, Sørensen S, Bakken KS Application of molecular genetics for diagnosing familial hypercholesterolemia in Norway: results from a family-based screening program. Semin Vasc Med. 2004;4(1):75–85. 15199436 10.1055/s-2004-822989

[43] Mak YT, Zhang J, Chan YS, Mak TW, Tomlinson B, Masarei JR, Pang CP Possible common mutations in the low density lipoprotein receptor gene in Chinese. Hum Mutat. 1998;11(Suppl 1):S310–S313. 10.1002/humu.1380110197 9452118

[44] Fouchier SW, Defesche JC, Umans-Eckenhausen MW, Kastelein JP The molecular basis of familial hypercholesterolemia in The Netherlands. Hum Genet. 2001;109(6):602–615. 11810272 10.1007/s00439-001-0628-8

[45] Zakharova FM, Damgaard D, Mandelshtam MY, Golubkov VI, Nissen PH, Nilsen GG, Stenderup A, Lipovetsky BM, Konstantinov VO, Denisenko AD, Vasilyev VB, Faergeman O Familial hypercholesterolemia in St-Petersburg: the known and novel mutations found in the low density lipoprotein receptor gene in Russia. BMC Med Genet. 2005;6(1):6. 15701167 10.1186/1471-2350-6-6 PMC551615

[46] Chiou K-R, Charng M-J Detection of mutations and large rearrangements of the low-density lipoprotein receptor gene in Taiwanese patients with familial hypercholesterolemia. Am J Cardiol. 2010;105(12):1752–1758. 20538126 10.1016/j.amjcard.2010.01.356

[47] Chang J-H, Pan J-P, Tai D-Y, Huang A-C, Li P-H, Ho H-L, Hsieh H-L, Chou S-C, Lin W-L, Lo E, Chang C-Y, Tseng J, Su M-T, Lee-Chen G-J Identification and characterization of LDL receptor gene mutations in hyperlipidemic Chinese. J Lipid Res. 2003;44(10):1850–1858. 12837857 10.1194/jlr.M200470-JLR200

[48] Sukhai MA, Craddock KJ, Thomas M, Hansen AR, Zhang T, Siu L, Bedard P, Stockley TL, Kamel-Reid S A classification system for clinical relevance of somatic variants identified in molecular profiling of cancer. Genet Med. 2016;18(2):128–136. 25880439 10.1038/gim.2015.47

[49] Nordestgaard BG, Chapman MJ, Humphries SE, Ginsberg HN, Masana L, Descamps OS, Wiklund O, Hegele RA, Raal FJ, Defesche JC, Wiegman A, Santos RD, Watts GF, Parhofer KG, Hovingh GK, Kovanen PT, Boileau C, Averna M, Borén J, Bruckert E, Catapano AL, Kuivenhoven JA, Pajukanta P, Ray K, Stalenhoef AFH, Stroes E, Taskinen M-R, Tybjærg-Hansen A; European Atherosclerosis Society Consensus Panel Familial hypercholesterolaemia is underdiagnosed and undertreated in the general population: guidance for clinicians to prevent coronary heart disease: consensus statement of the European Atherosclerosis Society. Eur Heart J. 2013;34(45):3478–3490. 23956253 10.1093/eurheartj/eht273 PMC3844152

[50] Dong C, Wei P, Jian X, Gibbs R, Boerwinkle E, Wang K, Liu X Comparison and integration of deleteriousness prediction methods for nonsynonymous SNVs in whole exome sequencing studies. Hum Mol Genet. 2015;24(8):2125–2137. 25552646 10.1093/hmg/ddu733 PMC4375422

[51] Jalali Sefid Dashti M, Gamieldien J A practical guide to filtering and prioritizing genetic variants. Biotechniques. 2017;62(1):18–30. 28118812 10.2144/000114492

[52] Metherell LA, Guerra-Assunção JA, Sternberg MJ, David A Three-dimensional model of human nicotinamide nucleotide transhydrogenase (NNT) and sequence-structure analysis of its disease-causing variations. Hum Mutat. 2016;37(10):1074–1084. 27459240 10.1002/humu.23046 PMC5026163

[53] Howard SR, Guasti L, Ruiz-Babot G, Mancini A, David A, Storr HL, Metherell LA, Sternberg MJ, Cabrera CP, Warren HR, Barnes MR, Quinton R, de Roux N, Young J, Guiochon-Mantel A, Wehkalampi K, André V, Gothilf Y, Cariboni A, Dunkel L IGSF10 mutations dysregulate gonadotropin-releasing hormone neuronal migration resulting in delayed puberty. EMBO Mol Med. 2016;8(6):626–642. 27137492 10.15252/emmm.201606250 PMC4888853

[54] Salvatori R, Radian S, Diekmann Y, Iacovazzo D, David A, Gabrovska P, Grassi G, Bussell A-M, Stals K, Weber A, Quinton R, Crowne EC, Corazzini V, Metherell L, Kearney T, Du Plessis D, Sinha AK, Baborie A, Lecoq A-L, Chanson P, Ansorge O, Ellard S, Trainer PJ, Balding D, Thomas MG, Korbonits M In-frame seven amino-acid duplication in *AIP* arose over the last 3000 years, disrupts protein interaction and stability and is associated with gigantism. Eur J Endocrinol. 2017;177(3):257–266. 28634279 10.1530/EJE-17-0293 PMC5510572

[55] David A, Razali R, Wass MN, Sternberg MJE Protein-protein interaction sites are hot spots for disease-associated nonsynonymous SNPs. Hum Mutat. 2012;33(2):359–363. 22072597 10.1002/humu.21656

[56] Ittisoponpisan S, Alhuzimi E, Sternberg MJE, David A Landscape of pleiotropic proteins causing human disease: structural and system biology insights. Hum Mutat. 2017;38(3):289–296. 27957775 10.1002/humu.23155 PMC5748329

