# Supplementary material for: Structural Biology Helps Interpret Variants of Uncertain Significance in Genes Causing Endocrine and Metabolic Disorders
Source: J Endocr Soc. 2018 Jun 13;2(8):842–54. doi: 10.1210/js.2018-00077 (PMC6041779; doi:10.1210/js.2018-00077)
Supplement: Supplemental Table S1 [file js.2018-00077.st1.doc]

**Structural biology aids in the interpretation of genetic variants of uncertain clinical significance in genes causing endocrine and metabolic disorders**

Sirawit Ittisoponpisan, Alessia David

Structural Bioinformatics Group, Department of Life Sciences, Imperial College London, London, SW7 2AZ, UK

**Supplementary Table S1** Description of variants presented as case studies. MAF, minor allele frequency reported in ExAC database; CH, Congenital hypothyroidism; FH, Familial hypercholesterolemia; FHH, Familial Hypocalciuric Hypercalcemia;

| **Gene** | **Amino acid change** | **Genetic variant**  **(HGVS)** | **dbSNP**  **(RefSNP)** | **MAF** | **PolyPhen2** | **SIFT** | **Mutation**  **Assessor** | **Condel** | **Structural analysis** | **Associated**  **condition** |
| --- | --- | --- | --- | --- | --- | --- | --- | --- | --- | --- |
| TSHR | p.Gly132Arg | NM_000369.2:  c.394G>C | [rs760874290](https://www.ncbi.nlm.nih.gov/variation/tools/1000genomes/?chr=14&from=81557414&to=81557414&gts=rs760874290&mk=81557414:81557414|rs760874290) | 0.00003 | Probably damaging | Tolerated | Neutral | Neutral | Damaging | CH |
| APOE | p.Leu46Pro | NM_000041.3:  c.137T>C | [rs769452](https://www.ncbi.nlm.nih.gov/variation/tools/1000genomes/?chr=19&from=45411110&to=45411110&gts=rs769452&mk=45411110:45411110|rs769452) | 0.00242 | Possibly damaging | Tolerated | Medium effect | Damaging | Damaging | FH |
| CASR | p.Gly36Arg | NM_000388.3:  c.106G>A | [rs193922420](https://www.ncbi.nlm.nih.gov/variation/tools/1000genomes/?chr=3&from=121973142&to=121973142&gts=rs193922420&mk=121973142:121973142|rs193922420) | NA | Probably damaging | Damaging | High | Damaging | Damaging | FHH |
| CASR | p.Gly143Arg | NM_000388.3:  c.427G>A | [rs769256610](https://www.ncbi.nlm.nih.gov/variation/tools/1000genomes/?chr=3&from=121976169&to=121976169&gts=rs769256610&mk=121976169:121976169|rs769256610) | 0.00008 | Probably damaging | Damaging | High | Damaging | Damaging | FHH |
| CASR | p.Cys568Gly | NM_000388.3:c.1702T>G | [rs1060502851](https://www.ncbi.nlm.nih.gov/variation/tools/1000genomes/?chr=3&from=122001053&to=122001053&gts=rs1060502851&mk=122001053:122001053|rs1060502851) | NA | Benign | Damaging | not available | Damaging | Damaging | FHH |
| LDLR | p.Cys329Tyr | NM_000527.4:  c.986G>A | [rs761954844](https://www.ncbi.nlm.nih.gov/SNP/snp_ref.cgi?type=rs&rs=761954844) | 0.00002 | Probably damaging | Damaging | not available | Damaging | Damaging | FH |
| LDLR | p.Cys284Ser | NM_000527.4:  c.850T>A | [rs879254693](https://www.ncbi.nlm.nih.gov/variation/tools/1000genomes/?chr=19&from=11218100&to=11218100&gts=rs879254693&mk=11218100:11218100|rs879254693) | NA | Benign | Damaging | not available | Damaging | Damaging | FH |
| LDLR | p.Ser499Pro | NM_000527.4:c.1495T>C | [rs879254921](https://www.ncbi.nlm.nih.gov/variation/tools/1000genomes/?chr=19&from=11224347&to=11224347&gts=rs879254921&mk=11224347:11224347|rs879254921) | NA | Benign | Damaging | Medium effect | Damaging | Damaging | FH |
| LDLR | p. Cys698Trp | NM_000527.4:c.2094C>G | [rs879255137](https://www.ncbi.nlm.nih.gov/variation/tools/1000genomes/?chr=19&from=11231152&to=11231152&gts=rs879255137&mk=11231152:11231152|rs879255137) | NA | Possibly damaging | Damaging | not available | Damaging | Damaging | FH |
